# Gammaherpesvirus Usurps Host IL-17 Signaling To Support the Establishment of Chronic Infection

**DOI:** 10.1128/mBio.00566-21

**Published:** 2021-04-06

**Authors:** C. N. Jondle, K. E. Johnson, C. Aurubin, P. Sylvester, G. Xin, W. Cui, A. R. Huppler, V. L. Tarakanova

**Affiliations:** a Department of Microbiology and Immunology, Medical College of Wisconsin, Milwaukee, Wisconsin, USA; b Cancer Center, Medical College of Wisconsin, Milwaukee, Wisconsin, USA; c Blood Research Institute, Versiti, Milwaukee, Wisconsin, USA; d Department of Pediatrics, Children’s Hospital of Wisconsin Research Institute, Medical College of Wisconsin, Milwaukee, Wisconsin, USA; National Cancer Institute; University of North Carolina, Chapel Hill

**Keywords:** IL-17 signaling, chronic infection, gammaherpesvirus, germinal center response

## Abstract

Gammaherpesviruses establish lifelong infection and are associated with a variety of cancers, including B cell lymphomas. These viruses manipulate the B cell differentiation process to establish lifelong infection in memory B cells. Specifically, gammaherpesviruses infect naive B cells and promote entry of both infected and uninfected naive B cells into germinal centers, where the virus usurps rapid proliferation of germinal center B cells to exponentially increase its cellular latent reservoir. In addition to facilitating the establishment of latent infection, germinal center B cells are thought to be the target of viral transformation. In this study, we have uncovered a novel proviral role of host interleukin 17A (IL-17A), a well-established antibacterial and antifungal factor. Loss of IL-17A signaling attenuated the establishment of chronic gammaherpesvirus infection and gammaherpesvirus-driven germinal center response in a route of inoculation-dependent manner. Further, IL-17A treatment directly supported gammaherpesvirus reactivation and *de novo* lytic infection. This study is the first demonstration of a multifaceted proviral role of IL-17 signaling.

## Abstract (precis)

Gammaherpesviruses establish lifelong infections in a majority of humans and are associated with B cell lymphomas. IL-17A is a host cytokine that plays a well-established role in the clearance of bacterial and fungal infections; however, the role of IL-17A in viral infections is poorly understood.

## INTRODUCTION

Gammaherpesviruses, such as Epstein-Barr virus (EBV) and Kaposi’s sarcoma-associated herpesvirus (KSHV), are ubiquitous pathogens that establish lifelong infection and are associated with multiple cancers, including B cell lymphomas (1). Gammaherpesviruses are DNA viruses that usurp a polyclonal germinal center response to amplify the latent viral reservoir in germinal center B cells and subsequently transition to a lifelong latency in memory B cells (2). In contrast to other virus infections, the robust germinal center response induced by gammaherpesvirus infection is unique in that germinal center B cells support the majority of the latent viral reservoir, especially during early stages of chronic infection (3, 4). Further, germinal center B cells are thought to be the target of viral transformation, as most EBV-positive B cell lymphomas are of germinal center or post-germinal-center origin (5). Importantly, host and viral mechanisms that facilitate or attenuate germinal center response during gammaherpesvirus infection remain poorly understood.

Interleukin 17A (IL-17A) is the founding member of the IL-17 cytokine family, which consists of IL-17A through IL-17F (6). IL-17A and IL-17F are the best characterized family members, they form covalent homodimers and heterodimers that bind and signal through a heterodimeric receptor complex of IL-17 receptor A (IL-17RA) and IL-17 receptor C (IL-17RC) (7–9). IL-17 cytokines were first associated with Th17 cells as IL-17A expression is the defining feature of the Th17 subset of CD4^+^ T cells (10, 11). While Th17 population is the best characterized source of IL-17, other T cell subsets and some innate immune populations can produce IL-17 family cytokines (12–14). IL-17A/F are important antimicrobial cytokines that are induced in response to and contribute to the clearance of multiple bacterial and fungal pathogens (15, 16). Persistently elevated IL-17A/F levels are also associated with several autoimmune diseases, including psoriasis, rheumatoid arthritis, and Crohn’s disease (17). In contrast, the role of IL-17 in viral infection is less understood. Overexpression of IL-17A increases vaccinia virus virulence, and endogenous IL-17 exacerbates corneal inflammation during herpes simplex virus 1 (HSV-1) infection without any effect on HSV-1 replication (18, 19). Similarly, while IL-17 does not alter acute HSV-2 replication in the female genital tract, it contributes to the maintenance of CD4^+^ tissue resident memory T cells (20) and supports survival of peripheral neurons during HSV-2 reactivation (21).

The role of IL-17 signaling during gammaherpesvirus infection has not been defined. Intriguingly, herpesvirus saimiri (HVS), a simian gammaherpesvirus, encodes a viral homologue of host IL-17, which functions similar to host IL-17A in culture (22, 23). Importantly, the role of viral IL-17 (vIL-17) in HVS infection of its natural host is not known. Because other gammaherpesviruses are not known to encode a viral homologue of IL-17A, it is intriguing to speculate that such IL-17-less gammaherpesviruses may usurp host IL-17A signaling. Indeed, in this study, we show a significant attenuation of chronic gammaherpesvirus infection and virus-driven germinal center response in the absence of IL-17RA, a critical IL-17A receptor of the host. Further, *ex vivo* stimulation of latently infected splenocytes or primary macrophages with recombinant IL-17A stimulated gammaherpesvirus reactivation and *de novo* lytic replication, respectively. This study highlights a novel and multifaceted proviral role of host IL-17 signaling during gammaherpesvirus infection.

## RESULTS

### IL-17RA signaling facilitates the establishment of chronic gammaherpesvirus infection.

Exquisite host specificity of human gammaherpesviruses EBV and KSHV and the intricate manipulation of B cell differentiation by these viruses significantly hinder studies of chronic infection. In contrast, genetically and biologically related murine gammaherpesvirus 68 (MHV68) offers a highly tractable experimental system to define virus-host interactions during chronic gammaherpesvirus infection of an intact host (24–26). Following infection of a naive animal, MHV68 acutely replicates at multiple anatomic locations, with lytic replication controlled by 12 days postinfection and the peak of viral latency observed at 16 days postinfection. Similar to EBV and KSHV, MHV68 does not encode a discernible IL-17 homologue. To define the role of IL-17A/F signaling during chronic gammaherpesvirus infection, wild-type mice or mice genetically deficient in IL-17 receptor A (IL-17RA^−/−^), a moiety critical for IL-17A and IL-17F signaling (9), were intranasally infected with MHV68. Parameters of MHV68 latency were assessed 16 days later in the spleen and peritoneal cavity, two major anatomic locations of latent MHV68 infection.

**FIG 1:**
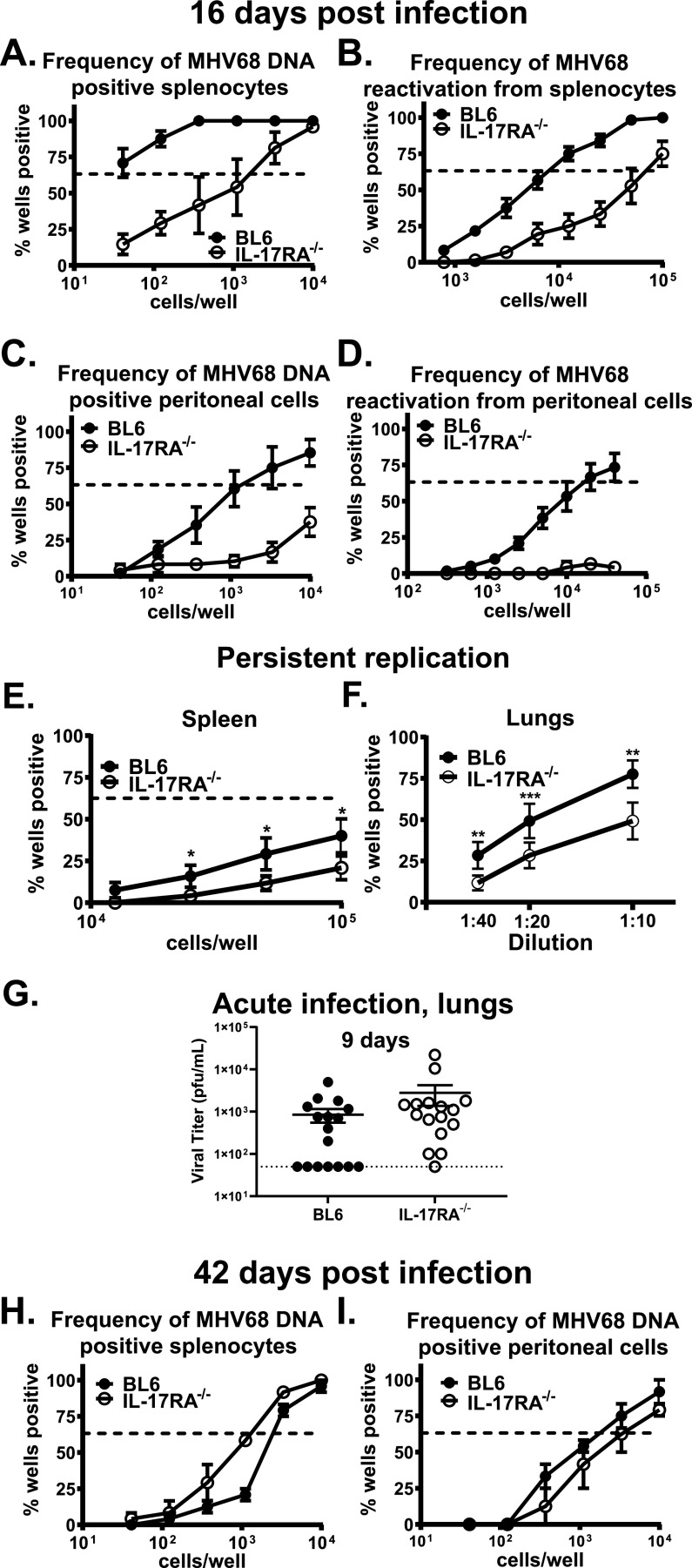
IL-17RA signaling facilitates the establishment of chronic gammaherpesvirus infection. BL6 and IL-17RA^−/−^ mice were intranasally infected with 1,000 PFU of MHV68. Splenocytes (A and B) and peritoneal cells (C and D) were harvested at 16 days postinfection and subjected to limiting dilution assays to determine the frequency of MHV68-positive cells (A and C) and the frequency of *ex vivo* viral reactivation (B and D). To determine the impact of IL-17RA deficiency on persistent MHV68 replication, lungs (E) and splenocytes (F) were physically disrupted and subjected to limiting dilution assays to determine the presence of preformed virus. (G). Acute MHV68 titers in lungs of mice with the indicated genotypes collected at 9 days postinfection. Each symbol represents the value for an individual lung. The dashed line represents the limit of detection. Splenocytes (H) and peritoneal cells (I) were harvested at 42 days postinfection and subjected to limiting dilution assays to determine the frequency of MHV68-positive cells. In the limiting dilution assays, the dashed line is drawn at 63.2%, and the *x* coordinate of intersection of this line with the sigmoid graph represents an inverse of the frequency of positive events. Each experimental group consists of three or four animals; data are pooled from two to six independent experiments. Means ± standard errors of the means (error bars) are shown. Values that are significantly different in this figure and subsequent figures are indicated by asterisks as follows: *, *P* < 0.05; **, *P* < 0.001; ***, *P* < 0.001.

The frequencies of MHV68 DNA-positive splenocytes and peritoneal cells were significantly decreased in IL-17RA^−/−^ mice (Fig. 1A and C; summary of all viral phenotypes in Table 1) along with decrease in the frequency of *ex vivo* reactivation (Fig. 1B and D). Low levels of persistently replicating, preformed MHV68 were further decreased in the lungs and spleens of IL-17RA^−/−^ mice compared to wild-type mice (Fig. 1E and F), and preformed lytic virus was undetectable in peritoneal cells of both groups (data not shown). The observed decrease in MHV68 latency and reactivation was not accompanied by the increased presence of gamma interferon (IFN-γ)-expressing MHV68-specific CD4 and CD8 T cells in IL-17RA^−/−^ mice (see Fig. S1A and B in the supplemental material). Further, acute MHV68 titers were similar in the lungs of BL6 and IL-17RA^−/−^ mice (Fig. 1G). Thus, attenuated establishment of chronic MHV68 infection in IL-17RA^−/−^ mice revealed an unexpected proviral role of IL-17RA signaling during the establishment of latent gammaherpesvirus infection.

**TABLE 1:** Frequency of cells harboring MHV68 genomes

Mouse strain	Route of infection	Cell population	dpi^*a*^	Avg frequency of MHV68 genome-positive cells^*b*^
BL6	Intranasal	Splenocytes	16	<1/40
IL-17RA^−/−^	Intranasal	Splenocytes	16	1/1,399

BL6	Intranasal	Peritoneal cells	16	1/1,300
IL-17RA^−/−^	Intranasal	Peritoneal cells	16	>1/10,000

BL6	Intranasal	Splenocytes	42	1/2,972
IL-17RA^−/−^	Intranasal	Splenocytes	42	1/1,275

BL6	Intranasal	Peritoneal cells	42	1/1,533
IL-17RA^−/−^	Intranasal	Peritoneal cells	42	1/3,319

BL6	Intraperitoneal	Splenocytes	16	1/160
IL-17RA^−/−^	Intraperitoneal	Splenocytes	16	1/119

BL6	Intraperitoneal	Peritoneal cells	16	1/160
IL-17RA^−/−^	Intraperitoneal	Peritoneal cells	16	1/149

a dpi, days postinfection.

b Average frequency of cells harboring MHV68 genomes. The average frequencies derived from two to six independent experiments are shown.

10.1128/mBio.00566-21.1 FIG S1 Lack of IL-17RA during gammaherpesvirus infection does not impact IFN-γ expression by T cells. BL6 and IL-17RA^−/−^ mice were infected as described in the legend to Fig. 1. (A and B) Splenic T cells were analyzed at 16 days postinfection. Virus-specific CD4^+^ T cells producing IFN-γ cells were defined as CD3^+^ CD4^+^ IFN-γ^+^ cells following restimulation with GP150 MHV68 peptide (A). Virus-specific IFN-γ-producing CD8^+^ T cells were defined as CD3^+^ CD8^+^ IFN-γ^+^ cells following restimulation with Orf6 MHV68 peptide (B). Means and standard errors are shown with each symbol representing the value for an individual animal. Download FIG S1, PDF file, 0.3 MB. Copyright © 2021 Jondle et al. 2021 Jondle et al. https://creativecommons.org/licenses/by/4.0/ This content is distributed under the terms of the Creative Commons Attribution 4.0 International license.

Following the peak of MHV68 splenic latency and reactivation observed at 16 days after intranasal infection, the latent viral reservoir in the spleen contracts and stabilizes by 42 days postinfection to be maintained for the life of the host. Concurrently, there is a significant reduction in viral reactivation to below detection levels. To determine the extent to which IL-17RA signaling alters long-term gammaherpesvirus infection, parameters of MHV68 latency were assessed at 42 days postinfection. Unlike that observed at the peak of MHV68 latency, there was no longer a difference in the frequency of MHV68 DNA-positive splenocytes and peritoneal cells between IL-17RA^−/−^ and wild-type mice (Fig. 1H and I). Further, MHV68 reactivation was below the level of detection in IL-17RA^−/−^ and wild-type mice (data not shown). Thus, while IL-17RA signaling promoted the establishment of peak levels of MHV68 chronic infection, the maintenance of long-term MHV68 latent reservoir was independent of IL-17RA signaling.

### Gammaherpesvirus infection stimulates IL-17 expression in diverse B and T cell populations.

**FIG 2:**
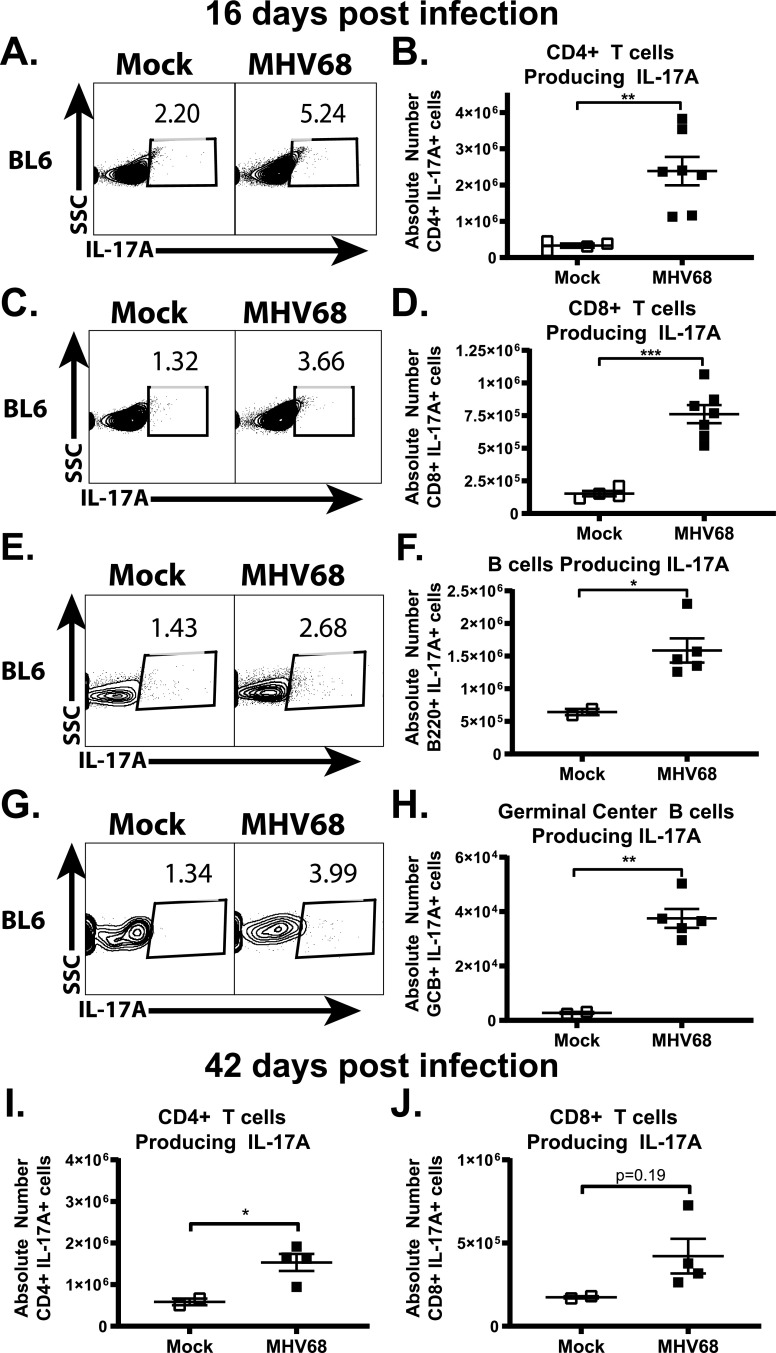
Gammaherpesvirus infection stimulates IL-17 expression in diverse B and T cell populations. BL6 mice were infected as described in the legend to Fig. 1, and the indicated splenic immune populations were analyzed at 16 and 42 days postinfection. Gating strategies are outlined in Fig. S2 in the supplemental material. MHV68-specific CD4^+^ T cells producing IL-17A cells were defined as CD3^+^ CD4^+^ IL-17A^+^ cells following restimulation with GP150 MHV68 peptide (A, B, and I). MHV68-specific IL-17A-producing CD8^+^ T cells were defined as CD3^+^ CD8^+^ IL-17A^+^ cells following restimulation with Orf6 MHV68 peptide (C, D, and J). Total splenic B cells producing IL-17A directly *ex vivo*, without additional restimulation, were defined as CD3^−^ B220^+^ IL-17A^+^ (E and F), while germinal center B cells producing IL-17A were defined as CD3^−^ B220^+^ CD95^+^ GL7^+^ IL-17A^+^ (G and H). Each symbol represents the value for an individual animal. Means and standard errors are shown, with statistical significance derived from Student’s *t* test. SSC, side scatter.

Given the well characterized role of Th17 CD4 T cells in the production of IL-17A along with reports of other immune cell populations capable of IL-17A expression (12–14, 27–30), IL-17A-producing immune populations were defined in wild-type mice at 16 days after MHV68 infection using gating strategies defined in Fig. S2. As expected and consistent with the proviral role of IL-17RA signaling, MHV68 infection triggered an increase in MHV68-specific CD4 T cell population expressing IL-17A following *ex vivo* restimulation with immunodominant MHV68 epitopes (Fig. 2A and B). Interestingly, MHV68-infected mice also showed an increase in the MHV68-specific CD8 T cells that expressed IL-17A following *ex vivo* restimulation with the MHV68 epitopes (Fig. 2C and D). While B cells are not a conventional source of IL-17A, we were surprised to find that MHV68-infected animals demonstrated an increased abundance of IL-17A-expressing splenic B cells (Fig. 2E and F) and, particularly, germinal center B cells (Fig. 2G and H), with the latter population not previously appreciated as the source of IL-17A. Unlike IL-17A expression by T cells that required *ex vivo* stimulation to become detectable, intracellular IL-17A was detectable in B cells immediately upon isolation from the spleens. In summary, MHV68 infection stimulated IL-17A expression by diverse immune cell populations at 16 days postinfection.

10.1128/mBio.00566-21.2 FIG S2 Flow cytometry gating strategies. Samples to be analyzed via flow cytometry were prepared and stained as described in Materials and Methods. Relevant cell populations were gated as follows. Singlets and lymphocytes were gated prior to CD3 gating. T cells were defined as CD3^+^ cells, while B220^+^ B cells were gated from the CD3-negative population. Having defined T cells and B cells, further gating occurred as follows. CD3^+^ T cells were gated into CD8^+^ T cells and further into IL-17A^+^ CD8^+^ T cells if the samples had been restimulated with MHV68 peptide. CD4^+^ T cells were also gated from the CD3^+^ population. If the sample had undergone *ex vivo* restimulated with MHV68 peptide, IL-17A-producing CD4^+^ T cells could be defined. T follicular helper cells (Tfh) were also defined from the CD4^+^ T cell population as CD4^+^ CXCR5^+^ PD-1^+^ cells. IL-17 expression was determined in either B220^+^ B cell population or following further gating on the B220^+^ GL7^+^ CD95^+^ germinal center B cells. Download FIG S2, PDF file, 1.4 MB. Copyright © 2021 Jondle et al. 2021 Jondle et al. https://creativecommons.org/licenses/by/4.0/ This content is distributed under the terms of the Creative Commons Attribution 4.0 International license.

Given similar parameters of long-term MHV68 infection in wild-type and IL-17RA^−/−^ mice (Fig. 1H and I), expression of IL-17A was defined at 42 days postinfection. The abundance of IL-17A-expressing CD4 T cells, as revealed by *ex vivo* restimulation with MHV68 immunodominant epitopes, continued to remain elevated in long-term infected compared to naive mice (Fig. 2I). However, the number of MHV68-specific CD4 T cells producing IL-17A at 42 days postinfection was significantly lower (*P* = 0.023) compared to the number of CD4 T cells producing IL-17A at 16 days postinfection (compare Fig. 2B and I). Further, there was no longer a statistically significant difference between naive and long-term infected mice in terms of CD8 T cells producing IL-17A following *ex vivo* restimulation with MHV68 peptides (Fig. 2J). Thus, a significant induction of multiple IL-17A producing immune populations observed at 16 days postinfection was subdued in long-term infected mice, consistent with the IL-17RA-independent nature of long-term MHV68 latency.

### IL-17RA signaling supports gammaherpesvirus-driven germinal center response during the establishment of viral latency.

**FIG 3:**
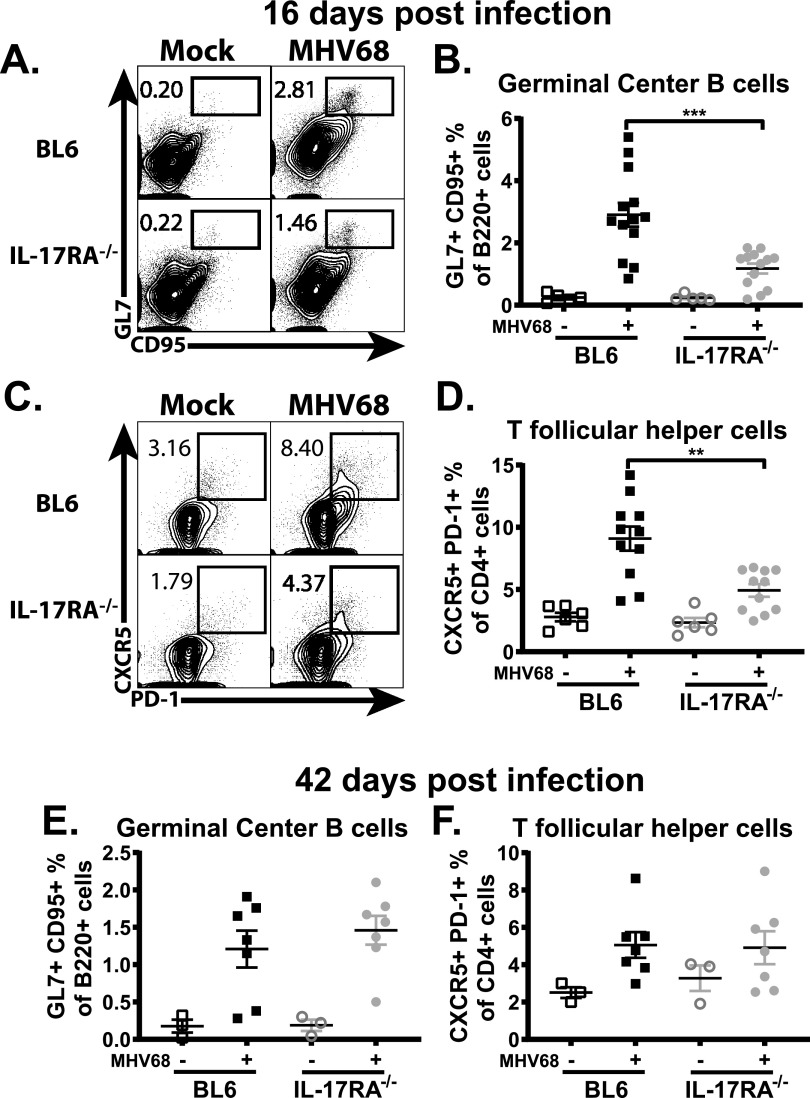
IL-17RA signaling supports gammaherpesvirus-driven germinal center response during the establishment of latency. BL6 and IL-17RA^−/−^ mice were infected as described in the legend to Fig. 1. The germinal center response was measured at 16 and 42 days postinfection, with germinal center B cells (A, B, and E) defined as B220^+^ GL7^+^ CD95^+^ cells and T follicular helper cells (C, D, and F) defined as CD3^+^ CD4^+^ CXCR5^+^ PD-1^+^ cells. Each experimental group consists of three or four animals. Data are pooled from two or three independent experiments. Means and standard errors of the means are shown.

Gammaherpesviruses usurp germinal center B cell differentiation in order to establish long-term latent infection in memory B cells. At the peak of MHV68 latency, the majority of latent viral reservoir is hosted by the germinal center B cells (3, 31), with CD4 T follicular helper cells (Tfh) playing a critical role in the MHV68-driven germinal center response (32). Having observed attenuated splenic MHV68 latency in IL-17RA^−/−^ mice, germinal center responses were assessed next. IL-17RA deficiency led to a significant decrease in the frequencies of germinal center B cell and Tfh populations at 16 days postinfection (Fig. 3A to D), consistent with the attenuated frequency of MHV68 positive splenocytes (Fig. 1A). In contrast, the more subdued germinal center response at 42 days postinfection was similar in wild type and IL-17RA^−/−^ mice (Fig. 3E and F). Thus, IL-17RA signaling supported MHV68-driven germinal center response during the establishment of MHV68 latency.

### IL-17RA signaling facilitates infection of germinal center/activated B cells.

**FIG 4:**
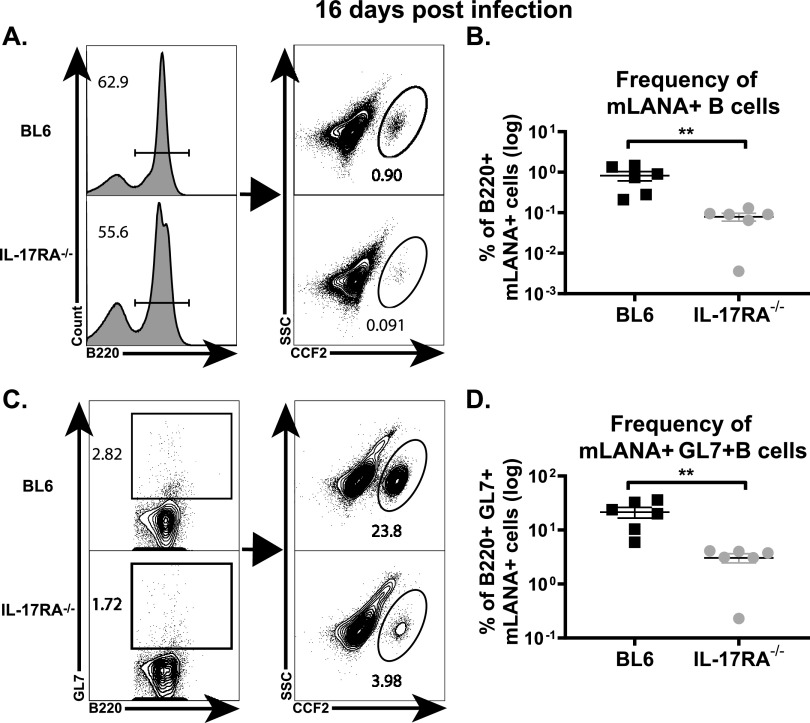
IL-17RA signaling supports gammaherpesvirus infection of B cells. BL6 and IL-17RA^−/−^ mice were intranasally infected with 1,000 PFU of MHV68.ORF73βla. At 16 days postinfection, splenocytes were isolated, pooled within each group, and stained for surface markers B220 and GL7. The cells were then loaded with CCF2-AM, the fluorescent β-lactamase substrate, for 1 h at room temperature Pooled splenocytes were examined for mLANA expression (β-lactamase activity) via flow cytometry, as indicated by the fluorescent signal of cleaved CCF2-AM. (A and B) The frequency of mLANA-positive (mLANA+) B cells as defined by B220^+^ cells. (C and D) The frequency of mLANA+ germinal center B cells as defined by B220^+^ GL7^+^ mLANA^+^ cells. Data are representative of three independent experiments.

Having observed a decrease in MHV68-driven germinal center response and the frequency of MHV68-positive splenocytes, the efficiency of germinal center B cell infection was examined next using a recombinant MHV68 that expresses a fused mLANA-β-lactamase protein in latently infected cells (33, 34). As expected, significantly fewer splenic B220^+^ B cells were also positive for β-lactamase activity in IL-17RA^−/−^ mice compared to BL6 mice (Fig. 4A and B). Interestingly, the frequency of β-lactamase- positive B220^+^ GL7^+^ B cells was also decreased approximately sixfold in IL-17RA^−/−^ spleens (Fig. 4C and D), indicating that IL-17RA signaling promotes the latent infection of activated/germinal center B cells.

### IL-17RA signaling is dispensable for the establishment of MHV68 latency following the intraperitoneal route of infection.

Viral and host requirements for the establishment of MHV68 latency are frequently dependent on the route of inoculation. While gammaherpesvirus entry through the host mucosa represents the natural route of infection, bypassing mucosal entry via direct inoculation into the body cavities or circulation has offered valuable insights into the biology of host and viral latency-defining factors that guide the course of natural infection. Having observed a significant attenuation of the MHV68 latent reservoir in IL-17RA^−/−^ mice following the intranasal route of infection, viral and host parameters were next examined following intraperitoneal inoculation.

**FIG 5:**
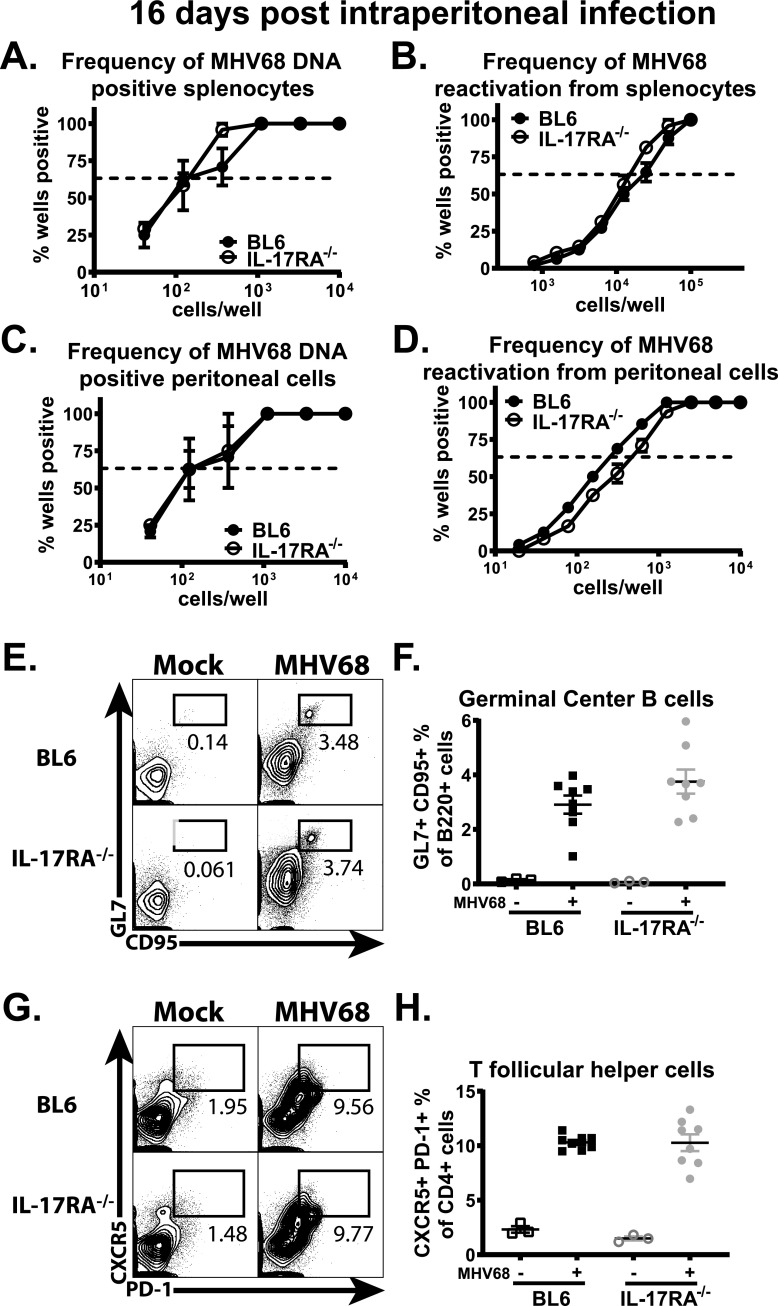
IL-17RA signaling is dispensable for the establishment of MHV68 latency following the intraperitoneal route of infection. BL6 and IL-17RA^−/−^ mice were intraperitoneally infected with 1,000 PFU of MHV68. Splenocytes (A and B) and peritoneal cells (C and D) were harvested at 16 days postinfection and subjected to limiting dilution assays to determine the frequency of MHV68-positive cells (A and C) and the frequency of *ex vivo* viral reactivation (B and D). The germinal center response was measured at 16 days postinfection, with germinal center B cells (E and F) defined as B220^+^ GL7^+^ CD95^+^ cells and T follicular helper cells (G and H) defined as CD3^+^ CD4^+^ CXCR5^+^ PD-1^+^ cells. Each experimental group consists of three or four animals. Data are pooled from two independent experiments. Means and standard errors of the means are shown.

Similar to that observed for other host and MHV68 virus mutants (35–37), intraperitoneal inoculation of wild-type and IL-17RA^−/−^ mice resulted in similar frequencies of MHV68 DNA-positive splenocytes and peritoneal cells (Fig. 5A and C) at 16 days postinfection. Similar frequencies of *ex vivo* reactivation were also observed in splenocytes (Fig. 5B) and peritoneal cells (Fig. 5D) of IL-17RA^−/−^ and wild-type mice. Further, intraperitoneal inoculation led to comparable frequencies of germinal center B cells (Fig. 5E and F) and T follicular helper cells (Fig. 5G and H). Thus, IL-17RA signaling selectively supported the establishment of chronic MHV68 infection following the intranasal, but not intraperitoneal, route of inoculation.

### Lack of IL-17RA expression reduces levels of MHV68-specific and self-directed class-switched antibodies stimulated by gammaherpesvirus infection.

**FIG 6:**
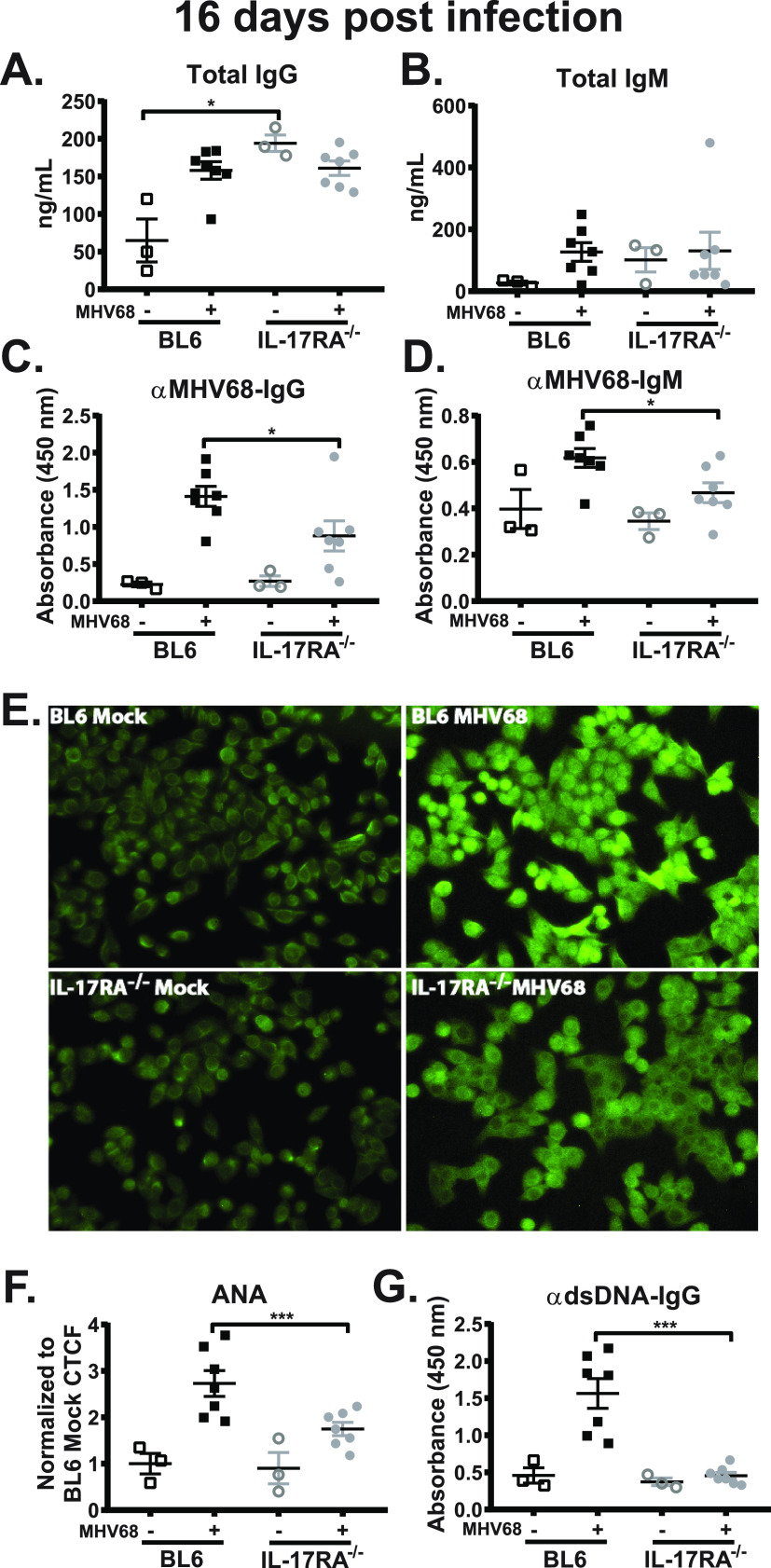
Lack of IL-17RA expression reduces levels of MHV68-specific and self-directed class-switched antibodies stimulated by gammaherpesvirus infection. Mice were infected as described in the legend to Fig. 1 or mock treated, and sera were collected at 16 days postinfection. (A to G) Sera were used to determine total IgG (A) and IgM (B), MHV68-specific IgG (C) and IgM (D), and dsDNA IgG (G) antibody titers. (E) Reactivity of mouse sera with Hep-2 monolayers (ANA) using anti-mouse IgG fluorescein isothiocyanate (FITC)-conjugated antibody for detection (representative images), corrected total cell fluorescence (CTCF) quantified in panel F. Data are pooled from two or three independent experiments, with each symbol representing value for an individual mouse. Means and standard errors of the means are shown. αMHV68 IgG, anti-MHV68 IgG.

Having observed a reduction in the germinal center response in IL-17RA^−/−^ mice infected via the intranasal route (Fig. 3A to D), humoral responses were measured next. Gammaherpesviruses uniquely induce a robust non-virus-specific B cell differentiation, leading to a rapid (albeit transient) increase in antibody titers against irrelevant and self-antigens (38), along with a delayed rise in virus-specific antibody titers (39). As demonstrated by other groups (40), baseline IgG levels were significantly elevated in IL-17RA^−/−^ mice (Fig. 6A) to the extent that MHV68 infection did not induce any further increase in total IgG serum levels compared to BL6 mice that showed significant elevation in circulating IgG induced by MHV68 infection. Further, total serum IgG and IgM levels were similar in MHV68-infected IL-17RA^−/−^ and BL6 mice (Fig. 6A and B). In contrast, MHV68-specific IgG and IgM titers were modestly decreased (1.6- and 1.3-fold, respectively) in IL-17RA^−/−^ mice compared to BL6 mice (Fig. 6C and D).

Gammaherpesviruses, including EBV and MHV68, induce a uniquely robust polyclonal increase in immunoglobulins directed against self-antigens and antigens of foreign species (38, 41). This induction of virus-nonspecific polyclonal antibody response forms the basis for the diagnostic assay, where high levels of antibodies directed against horse red blood cells are indicative of a recent EBV infection in humans (42). To capture the breadth of self- and foreign-antigen-directed antibody response induced by MHV68, sera from mock- and MHV68-infected mice were subjected to a clinical assay used in the diagnosis of autoimmune diseases (antinuclear antibody or ANA) (41). MHV68-infected IL-17RA^−/−^ mice showed significantly less overall self-antigen reactivity compared to BL6 mice (Fig. 6E and F). Interestingly, the pan-cellular staining pattern produced by sera from infected BL6 mice appeared to be modified when sera from IL-17RA^−/−^ mice were used, the latter producing decreased intensity of nuclear staining (Fig. 6F). To determine potential reason for the decreased nuclear staining, anti-double-stranded DNA (anti-dsDNA) antibodies were measured. In contrast to a significant increase in the anti-dsDNA titers in MHV68-infected BL6 mice, anti-dsDNA titers remained at baseline levels in MHV68-infected IL-17RA^−/−^ mice (Fig. 6G). Thus, IL-17RA signaling promoted both virus-specific and self-directed B cell differentiation, particularly antibody response against dsDNA stimulated by MHV68 infection.

### IL-17RA signaling facilitates *de novo* lytic infection and MHV68 reactivation.

**FIG 7:**
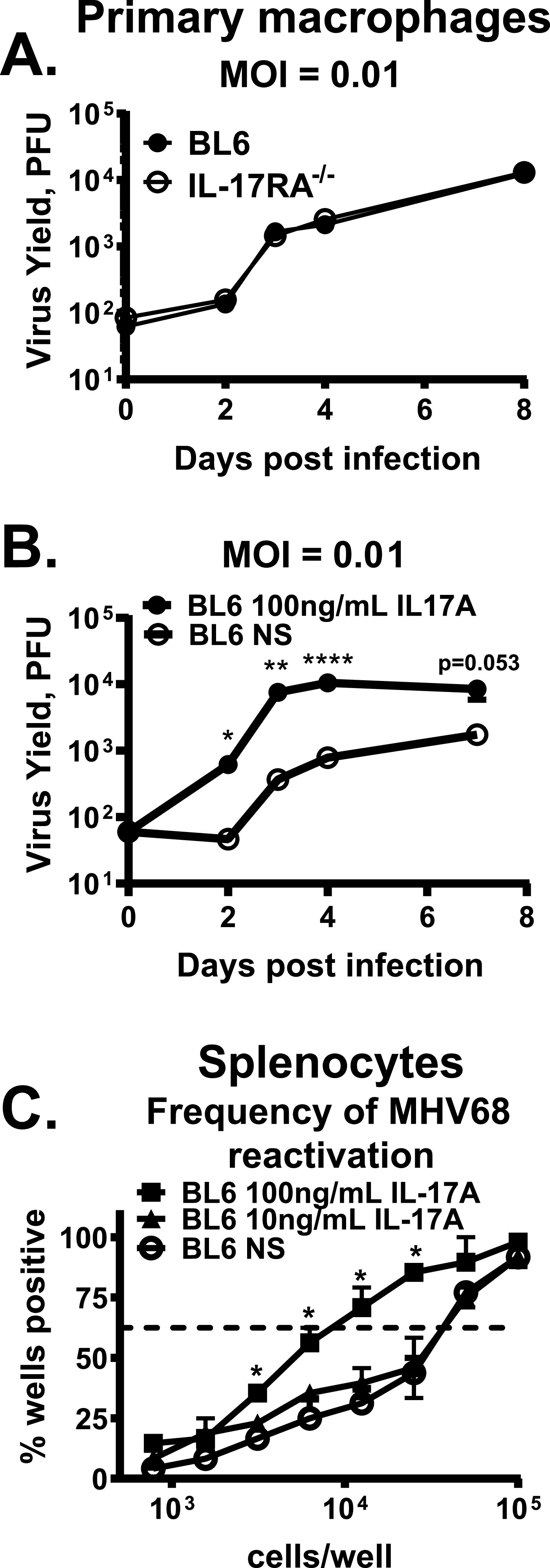
IL-17RA signaling facilitates *de novo* lytic MHV68 infection and reactivation. (A and B) Bone marrow-derived macrophages (BMDM) were generated from BL6 and IL-17RA^−/−^ mice and infected at 0.01 PFU/cell. MOI, multiplicity of infection. In panel B, BL6 macrophages were treated with 100 ng/ml of recombinant IL-17A or vehicle control added to the infected cultures immediately after viral adsorption for the remainder of the experiment, without any replenishment. Viral titers were determined at indicated time postinfection (A and B). (C) The frequency of *ex vivo* reactivation was determined using splenocytes collected from BL6 mice at 16 days after MHV68 infection. Reactivating cultures were treated with vehicle control (not stimulated [NS]), 10 ng/ml or 100 ng/ml of recombinant IL-17A. Data are pooled from two or three independent experiments. Differences in reactivation between not stimulated and 100 ng/ml IL-17A-stimulated groups are significant, with *P* = 0.0021. Means and standard errors of the means are shown.

Having observed proviral effects of IL-17RA signaling *in vivo*, we next wanted to determine the extent to which IL-17A directly promotes MHV68 replication and reactivation. IL-17RA receptor is ubiquitously expressed, including by bone marrow derived macrophages. MHV68 lytic titers were similar in IL-17RA^−/−^ and BL6 primary macrophages (Fig. 7A), indicating that MHV68 does not rely on IL-17RA for viral entry or subsequent stages of the replication cycle. However, addition of exogenous IL-17A enhanced MHV68 replication in primary BL6 macrophages (Fig. 7B), indicating that at least some of proviral effects of IL-17RA signaling observed *in vivo* may be mediated via direct effects on infected cells. To determine the extent to which proviral effects of IL-17A were cell type specific, reactivation of MHV68 was assessed in BL6 splenocytes harvested from latently infected mice and stimulated with increasing concentrations of recombinant IL-17A *ex vivo*. There was a significant increase in the frequency of viral reactivation following *ex vivo* stimulation with 100 ng/ml of recombinant IL-17A (Fig. 7C). Given that plasma cells support the majority of MHV68 reactivation from wild-type splenocytes (43) and ubiquitous expression of IL-17RA, these data suggest that IL17A proviral effects on MHV68 infection are not restricted to a single cell type.

## DISCUSSION

In this study, we uncovered an unanticipated proviral role of IL-17A/F signaling pathway during the establishment of gammaherpesvirus latency. Genetic deficiency of IL-17RA, the classical IL-17A/F receptor, produced a significant attenuation of peak gammaherpesvirus latency and gammaherpesvirus-driven germinal center response, a B cell differentiation stage that is key for both viral latency and lymphomagenesis. Results of our study and the fact that HVS, a simian gammaherpesvirus, encodes a functional viral IL-17 (22, 23) suggests that the gammaherpesvirus−IL-17 cross talk operates across species. Other gammaherpesviruses, including EBV and KSHV, do not encode a discernible IL-17 homologue, suggesting that these gammaherpesviruses, similar to MHV68, may usurp host IL-17. In support of this hypothesis, EBV-positive infectious mononucleosis patients display a significant increase in IL-17A-producing CD4^+^ T cells, an increase that persists at least 1 month following the resolution of clinical symptoms (44). This clinical observation in conjunction with our study opens the possibility that existing FDA-approved IL-17A neutralizing antibodies and other IL-17-targeting therapies could be repurposed for the control of gammaherpesvirus infection in susceptible patients.

### How might IL-17-less gammaherpesviruses induce host IL-17?

While the IL-17 cytokine family was initially associated with Th17 cells (10, 11), many other cell types such as γδ T cells (14), αβ innate cells (30), CD8^+^ T cells, natural killer T (NKT) cells (12), natural killer (NK) cells, group 3 innate lymphoid cells (ILC3s) (13), and even B cells (28, 29) can express IL-17. We also observed heterogenous immune populations expressing IL-17A in MHV68-infected animals, including germinal center B cells, a population that has not yet been identified as producers of IL-17A. Various immune cell types produce IL-17A downstream of differing combinations of IL-1, IL-6, IL-21, IL-23, and transforming growth factor β (TGF-β) via the transcription factor RORγt (15, 45). Interestingly, B cell production of IL-17A is independent of those factors, at least in the context of parasitic infection (28).

While IL-23 induces IL-17A production in many experimental systems (27, 46), MHV68 infection of IL-23p19^−/−^ mice surprisingly showed no difference in IL-17A-producing CD4^+^ T cells (data not shown), indicating that IL-23 is not critical for IL-17A induction during MHV68 infection. IL-1 is another cytokine that can stimulate IL-17A expression (47, 48). We showed an attenuated germinal center response in MHV68-infected IL-1R1^−/−^ mice (49), suggesting that IL-1 signaling may contribute to IL-17A expression induced by MHV68. Other possible facilitators of IL-17A expression include IL-6, as it is increased during MHV68 infection (50), or IL-21, as IL-21 signaling is required for efficient establishment of MHV68 latency and germinal center response in infected mice (51). In addition to classical inducers of IL-17A expression, novel mechanisms might stimulate IL-17A expression in germinal center B cells, potentially including viral factors, as germinal center B cells host the majority of the latent viral reservoir at the peak of viral latency.

### Why is IL-17 proviral for MHV68?

Classically, IL-17 cytokines facilitate clearance of multiple bacterial and fungal pathogens (15, 52). During infections with extracellular or intracellular bacterial pathogens, such as Klebsiella pneumoniae and Francisella tularensis, IL-17 facilitates the recruitment of neutrophils and production of antimicrobial peptides (52). Similar to its role in bacterial infection, IL-17-driven expression of antimicrobial peptides is important for the clearance of fungal infections, such as Candida albicans (53). In contrast, the role of IL-17 cytokines during viral infections is less understood. Host IL-17 promoted vaccinia virus dissemination and inflammatory disease in a mouse model of atopic dermatitis, and transgenic expression of IL-17 by recombinant vaccinia virus promoted its virulence in wild-type mice (18, 54). Similarly, IL-17 enhanced corneal inflammation and stromal keratitis during HSV-1 infection without an effect on viral replication (19, 55). In contrast, IL-17 family cytokines plays a protective role during other viral infections by inhibiting rhinovirus replication (56) as well as enhancing Th1 immunity in the female genital tract (20) and survival of peripheral neurons during HSV-2 infection (21). These contradicting observations indicate that unlike bacterial and fungal infections where IL-17 uniformly promotes pathogen clearance, the role of IL-17 during viral infection is more nuanced.

Gammaherpesviruses are unique in that they induce a robust germinal center response, which supports a majority of the latent viral reservoir (3, 4). These viruses also drive an increase in polyclonal immunoglobulins directed against nonrelevant antigens from foreign species and even self-directed antigens (38, 41). Interestingly, IL-17RA signaling has been found to promote the germinal center response and the development of antibodies driven by self-antigens in autoimmune mouse models (57, 58), in part by orchestrating the localization of T follicular helper cells into the germinal center light zone (59). In humans, IL-17RA is expressed on germinal center B cells and is thought to promote the germinal center B cell migration. Further, IL-17A stimulation of primary B cells from non-Hodgkin lymphomas promoted proliferation *in vitro*, while stimulation also enhanced tumor proliferation and angiogenesis *in vivo* (60). These data indicate that the impact of IL-17A signaling is conserved across species and highlights the significant ways in which IL-17A signaling could impact gammaherpesvirus infection and, possibly, lymphomagenesis. Not surprisingly, gammaherpesviruses may usurp some of the physiological host IL-17A functions during B cell differentiation, especially if the virus lacks its own IL-17A homologue. The fact that EBV-positive infectious mononucleosis patients have a significant increase in IL-17A-producing CD4^+^ T cells for at least a month following symptoms (44) supports the idea that gammaherpesviruses that lack an IL-17A homologue usurp host IL-17A signaling to promote infection.

Interestingly, the proviral effects of IL-17A signaling on MHV68 infection, including germinal center response, were selective for the intranasal, but not intraperitoneal, route of inoculation. This observation is not unprecedented, as other host and MHV68 mutants share the same phenotype. For example, MHV68-encoded M2 protein, while playing a minimal role during acute replication, is critical for the establishment of splenic latency following intranasal, but not intraperitoneal, infection (35). Further, B cell-specific deficiency of STAT3, while leading to profound attenuation of MHV68 peak latent splenic reservoir following intranasal inoculation, presents with a significantly more modest viral phenotype following intraperitoneal infection (36). Interestingly, we also observed that IL-17RA deficiency had no effect on the germinal center responses driven by chronic infection with lymphocytic choriomeningitis virus (LCMV) that was inoculated via the intraperitoneal route (data not shown). Given the critical role of STAT3 and MHV68 M2 in the regulation of B cell biology and differentiation, it is conceivable that the route of infection qualitatively alters the nature of the B cell differentiation induced and manipulated by MHV68 infection. One possibility could be the difference in mucosa-localized versus peritoneal cavity-based antigen-presenting cells that polarize the adaptive immune response, including B cell differentiation, during early stages of MHV68 infection. Consistent with this possibility, we and others have shown that MHV68 preferentially infects peritoneal macrophages following the intraperitoneal, but not intranasal, route of inoculation (61–63). Given that the natural route of gammaherpesvirus infection involves the mucosal interface, it will be important to define the differential host responses initiated at the mucosa versus body cavities during MHV68 infection to decipher the biology of viral and host mutants that selectively support the establishment of MHV68 latency following mucosal inoculation.

In addition to the possible effects on MHV68-driven B cell differentiation, we show that recombinant IL-17A stimulated MHV68 replication in primary macrophages and MHV68 reactivation from splenocytes, with the latter primarily supported by the plasma cells (43). This, combined with the ubiquitous expression of IL17RA, suggests that the proviral role of IL-17A signaling during gammaherpesvirus infection is not limited to a single cell type. In support of this idea, HVS, the only gammaherpesvirus known to encode viral IL-17, is T cell tropic. Interestingly, lytic MHV68 titers were not decreased in the lungs of acutely infected IL-17RA^−/−^ mice. Because epithelial cells represent the majority of MHV68-infected cells in the lungs (64, 65), this result also suggests that proviral effects of the IL-17RA signaling are not pervasive.

Finally, we have found similar frequencies of latently infected splenocytes and peritoneal cells in long-term infected BL6 and IL17RA^−/−^ mice, along with decreased abundance of IL-17A-producing T cells. Infection of developing B cells in the bone marrow contributes to the maintenance of long-term, but not peak, MHV68 latent reservoir (66, 67), a process that may be independent of IL-17A signaling. However, it is also possible that diminished expression of IL-17A is no longer sufficient to influence the parameters of long-term infection. In the latter possibility, it is tempting to speculate that physiological or pathological conditions resulting in increased IL-17A production *in vivo* may also promote gammaherpesvirus reactivation. IL-17A is strongly associated with certain autoimmune diseases, such as psoriasis, rheumatoid arthritis, and Crohn’s disease, with FDA-approved inhibitors of IL-17A used in treatment of some of these conditions (15, 17). Thus, in the future, it will be interesting to determine the extent to which chronic EBV and KSHV infections in these patients are modulated by IL-17A-targeting therapy.

## MATERIALS AND METHODS

### Animals used.

All experimental manipulations of mice were approved by the Institutional Animal Care and Use Committee of the Medical College of Wisconsin (MCW) (AUA971). C57BL/6J mice were obtained from The Jackson Laboratories (Bar Harbor, ME). IL-17RA^−/−^ mice were provided by Amgen (68). All mice were housed and bred in a specific-pathogen-free facility at MCW. Both male and female mice were used with no gender-specific phenotypes noted.

### Infections.

Between 6 and 10 weeks of age, mice were either intranasally or intraperitoneally inoculated with 1,000 PFU of MHV68 (WUMS) diluted in sterile serum-free Dulbecco’s modified Eagle’s medium (DMEM) (15 μl/mouse for intranasal, 300 μl/mouse for intraperitoneal), under light anesthesia. MHV68 viral stock was prepared, and the titers of the virus on NIH 3T12 cells were determined. The spleen and peritoneal cells were harvested from euthanized mock-treated and MHV68-infected animals at indicated times postinfection. Mice were euthanized by CO_2_ inhalation from a compressed gas source in a nonovercrowded chamber. Mice were bled prior to euthanasia via the submandibular route, and serum was isolated using BD Microtainer blood collection tubes (Becton, Dickinson and Company, Franklin Lakes, NJ). For acute studies, lungs were harvested at 9 days postinfection, and viral titers were measured as previously described (69).

### Limiting dilution assays.

The frequency of virally infected cells (cells harboring viral DNA) was determined by limiting dilution PCR analysis, while the frequency of *ex vivo* reactivation to identify cells capable of producing infectious virus was determined by limiting dilution assay as previously described (37). Briefly, to determine the frequency of cells reactivating virus *ex vivo*, serial twofold dilutions of splenocytes or peritoneal cell suspensions harvested from infected mice were plated onto monolayers of mouse embryonic fibroblasts (MEF) immediately following harvest, at 24 replicates per dilution. In order to control for any preformed infectious virus, twofold serial dilutions of mechanically disrupted lungs, splenocytes, or peritoneal cells were plated as described above. MHV68 was allowed to reactivate from primary cells, and virus was further amplified within the same well via subsequent replication in MEF. At 21 days postplating, all replicates and dilutions were scored in a binary fashion for the presence of live fibroblasts (no viral reactivation/replication) or absence of such (dead fibroblasts as a result of cytopathic effect driven by lytic replication). Because primary MEF were used to amplify the virus, the sensitivity of limiting dilution reactivation assay was below a single PFU of MHV68 defined using 3T12-based plaque assay. Because the endpoint of viral amplification in MEF was measured, the limiting dilution reactivation assay was not susceptible to variability of titers released from primary cells upon viral reactivation *ex vivo* (31).

### Flow cytometry.

Single cell suspensions of splenocytes and peritoneal cells from mock-infected and infected mice were prepared in fluorescence-activated cell sorting (FACS) buffer (phosphate-buffered saline [PBS] plus 2% fetal calf serum and 0.05% sodium azide) at 1 × 10^7^ nucleated cells/ml. A total of 1 × 10^6^ cells were treated with Fc block (24G2) prior to extracellular staining with an optimal antibody concentration for 30 min on ice. To determine IL-17A production in B cells, single cell suspensions of splenocytes were treated with Fc block and directly stained for extracellular markers, prior to intracellular staining for IL-17A. To intracellularly stain, cells were fixed and permeabilized using the BD Cytofix/Cytoperm kit (catalog no. 554714; Fisher Scientific, Hampton, NH). The cells were then intracellularly stained with an optimal antibody concentration for 30 min on ice. For T cell phenotyping, single cell suspensions of splenocytes and peritoneal cells were plated at 6 × 10^6^ nucleated cells/ml in a 96-well plate and stimulated with relevant virus-specific peptides and 10 μg/ml Brefeldin A (catalog no. 420601; BioLegend, San Diego, CA) in DMEM with 10% fetal bovine serum (FBS) for 6 h at 37°C. For virus-specific CD4^+^ T cells, 2.5 μg/ml of an MHV68-specific viral peptide GP150 (70) (GenScript, Piscataway, NJ) was utilized. IL-17A expression by MHV68-specific CD8 T cells was measured following stimulation with 10 μg/ml of ORF6 and ORF61 viral peptides (Thermo Fisher Scientific, Waltham, MA). B cells expressing IL-17A were detected directly *ex vivo*, without additional restimulation. Following restimulation, cells were washed in FACS buffer before treatment with Fc block (24G2) prior to extracellular staining with an optimal antibody concentration for 30 min on ice. After extracellular staining, the cells were intracellularly stained using optimal antibody concentrations for 30 min on ice as described above. Data acquisition was performed on a LSR II flow cytometer (BD Biosciences, San Jose, CA) and analyzed using FlowJo software (Tree Star, Ashland, OR). The following antibodies were purchased from BioLegend (San Diego, CA) for use in this study: CD3 (17A2), CD4 (RM4-5), CD8a (53-7.3), CD95 (Jo2), PD-1 (29f.1A12), B220 (RA3-6B2), GL7 (GL-7), IL-4 (11B11), and IL-17A (TC11-18H10.1). CXCR5 (2G8) was purchased from BD Pharmingem (San Jose, CA). Compensation controls were done using OneComp eBeads (Thermo Fisher Scientific, Waltham, MA). Briefly, a negative control (beads alone) was used to establish a baseline PMT (photomultiplier tube) voltage and fluorescent background. Positive controls for each fluorochrome (beads with a single fluorochrome) are used to establish spill-over for the individual fluorochrome into the other channels being used. PMT values are adjusted for each fluorochrome to minimize spill-over. Gating strategies are displayed in Fig. S2 in the supplemental material.

### MHV68.ORF73βla studies.

Mice were infected intranasally with 1,000 PFU MHV68.ORF73βla. Mice were euthanized at 16 days postinfection, with splenocytes pooled from each group (three mice/group). Cells (2 × 10^7^) from each group were Fc blocked and then stained with B220 and GL7 for 30 min on ice as described above. The cells were washed twice and loaded with CCF2-AM (GeneBLAzer kit; ThermoFisher Scientific, Waltham, MA) at room temperature for 1 h. The cells were then washed twice and suspended in FACS buffer prior to analysis via flow cytometry.

### Enzyme-linked immunosorbent assay (ELISA).

Total, MHV68-specific, and dsDNA immunoglobulin levels were determined as previously described (49). Briefly, Nunc Maxisorp plates (Fisher Scientific, Pittsburg, PA) were coated with antigen of interest, either anti-IgG (heavy and light) or anti-IgM antibodies (Jackson ImmunoResearch, West Grove, PA), UV-irradiated MHV68 virus stock in PBS (740,000 microjoules/cm^2^ × 2) (Stratalinker UV Crosslinker 1800; Agilent Technologies, Santa Clara, CA), or dsDNA from Escherichia coli (12.5 μg/ml; Sigma-Aldrich, St. Louis, MO) overnight at 4°C. Plates were washed with PBS containing Tween (PBS-Tween) (0.05%) and blocked for 1 h with PBS-Tween (0.05%)-BSA (3%), incubated with fivefold serial dilutions of serum in PBS-Tween (0.05%)-BSA (1.5%) for 2 h and then washed with PBS-Tween (0.05%). Bound antibody was detected with horseradish peroxidase (HRP)-conjugated goat anti-mouse total IgG (heavy and light chain [H+L]) or IgM (Jackson ImmunoResearch, West Grove, PA) using 3,3′,5,5′-tetramethylbenzidine substrate (Life Technologies, Gaithersburg, MD). HRP enzymatic activity was stopped by the addition of 1 N HCl (Sigma-Aldrich, St. Louis, MO), and the absorbance was read at 450 nm on a model 1420 Victor^3^V Multilabel plate reader (PerkinElmer, Waltham, MA).

### ANA panels.

Antinuclear antibodies were assessed with an antinuclear antibody (ANA) test kit (Antibodies Inc., Davis, CA). Following the manufacturer’s protocol, serum was diluted (1:40 in PBS) and incubated over slides coated with fixed HEp-2 cells. Following serum incubation, the slides were rinsed and stained with anti-mouse IgG labeled with Alexa Fluor 488 (H+L) (ThermoScientific, Waltham, MA). Fluorescent images were captured using NIS Elements software. Corrected fluorescence was quantified using ImageJ software from a randomly chosen field of ∼20 cells in each sample.

### Primary cell isolation, viral infection, and cell treatment.

Bone marrow was harvested from male and female mice between 3 and 10 weeks of age. Primary bone marrow-derived macrophages were generated as previously described (71). Bone marrow-derived macrophages were infected with MHV68 at indicated multiplicity of infection (MOI) for 1 h to allow for adsorption and washed two or three times with PBS prior to medium replenishment. For IL-17A (catalog no. 421-ML-025; R&D Systems, Inc., Minneapolis, MN) stimulation experiments, IL-17A was added to infected macrophage cultures immediately following virus adsorption or immediately after isolation of splenocytes, just prior to plating the cells on MEF monolayers. IL-17A was not replenished during either assay.

### Statistical analyses.

Statistical analyses were performed using Student’s *t* test (Prism; GraphPad Software, Inc.).
