# Supplementary material for: Gammaherpesvirus Usurps Host IL-17 Signaling To Support the Establishment of Chronic Infection
Source: mBio. 2021 Apr 6;12(2):e00566-21. doi: 10.1128/mBio.00566-21 (PMC8092251; doi:10.1128/mBio.00566-21)

Supplemental Figure 1

16 days post infection

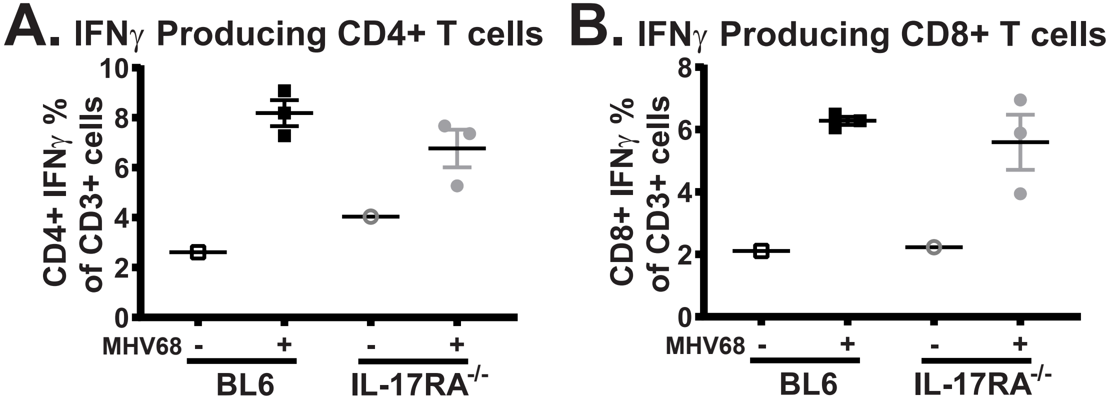

Supplement: FIG S1 [file mBio.00566-21-sf001.pdf]
