# Supplementary figures and images for: Gammaherpesvirus Usurps Host IL-17 Signaling To Support the Establishment of Chronic Infection
Source: mBio. 2021 Apr 6;12(2):e00566-21. doi: 10.1128/mBio.00566-21 (PMC8092251; doi:10.1128/mBio.00566-21)

# Supplemental Figure 2

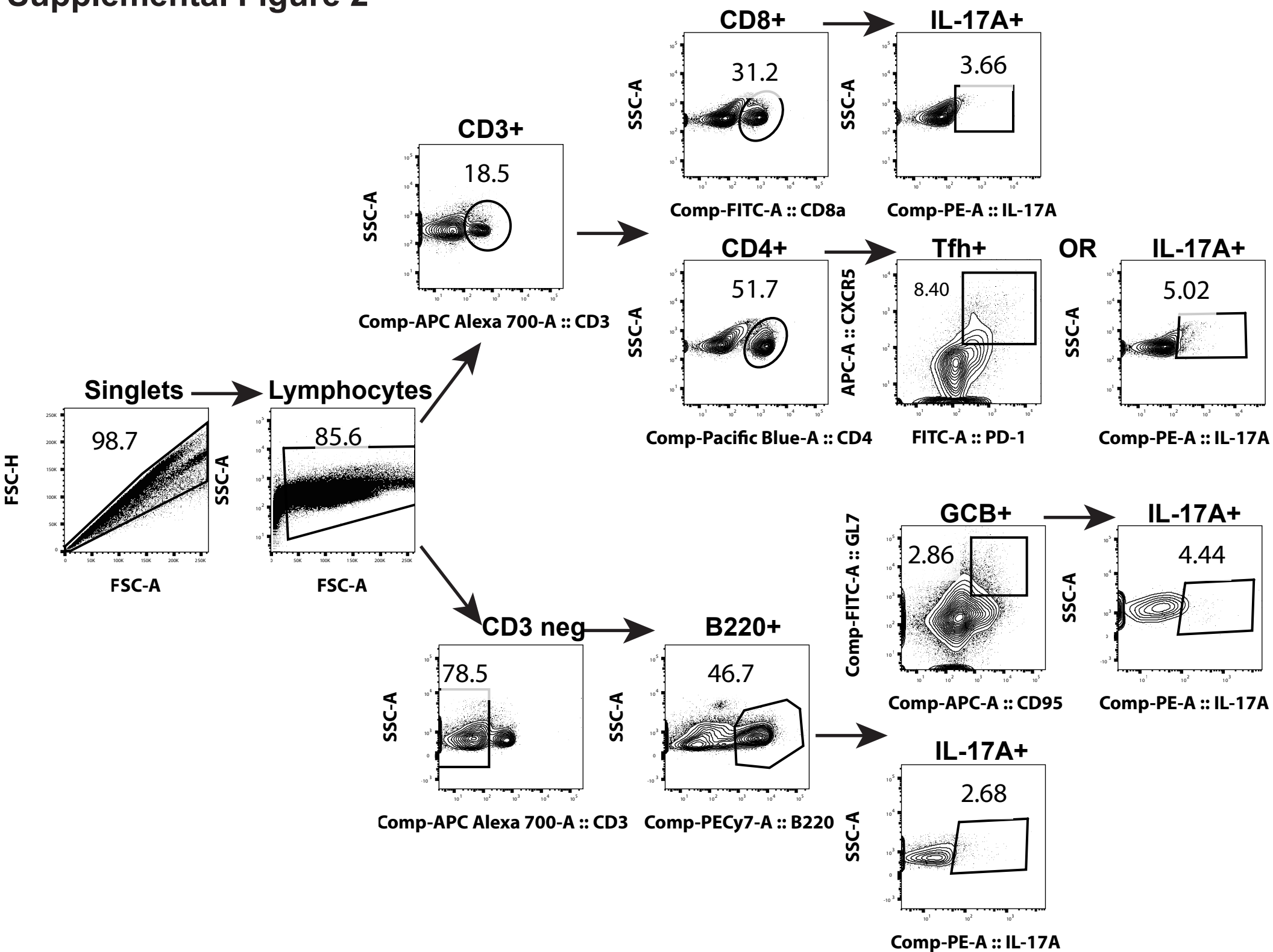

Supplement: FIG S2 [file mBio.00566-21-sf002.pdf]
